# An Electrochemical Sensor for Detection of Lead (II) Ions Using Biochar of Spent Coffee Grounds Modified by TiO_2_ Nanoparticles

**DOI:** 10.3390/molecules29235704

**Published:** 2024-12-03

**Authors:** Zaiqiong Liu, Yiren Xu, Xurundong Kan, Mei Chen, Jingyang Dai, Yanli Zhang, Pengfei Pang, Wenhui Ma, Jianqiang Zhang

**Affiliations:** 1 International Union Laboratory of China and Malaysia for Quality Monitoring and Evaluation of Agricultural Products in Yunnan, School of Biology and Chemistry, Pu’er University, Pu’er 665000, China; lzq15025110725@126.com (Z.L.); xuyiren@peu.edu.cn (Y.X.); kanxurundong@163.com (X.K.); mchen1988@126.com (M.C.); 18988150410@163.com (J.D.); 2 School of Chemistry and Environment, Yunnan Minzu University, Kunming 650500, China; yanli.zhang@yahoo.com (Y.Z.); pfpang@ynni.edu.cn (P.P.); 3 School of Engineering, Yunnan University, Kunming 650500, China

**Keywords:** coffee grounds-derived biochar, TiO_2_ nanoparticles, lead ions electrochemical sensor

## Abstract

Toxic heavy metal ions, such as lead ions, significantly threaten human health and the environment. This work introduces a novel method for the simple and sensitive detection of lead ions based on biochar-loaded titanium dioxide nanoparticles (BC@TiO_2_NPs) nanocomposites. Eco-friendly biochar samples were prepared from spent coffee grounds (500 °C, 1 h) that were chemically activated with TiO_2_ nanoparticles (150 °C, 24 h) to improve their conductivity. Structural characterizations showed that BC@TiO_2_NPs have a porous structure. The BC@TiO_2_NPs material was evaluated for lead ion determination by assembling glassy carbon electrodes. Under optimal conditions, the sensor was immersed in a solution containing the analyte (0.1 M NaAc-HAc buffer, pH = 4.5) for the detection of lead ions via differential pulse voltammetry. A linear dynamic range from 1 pM to 10 μMwas achieved, with a detection limit of 0.6268 pM. Additionally, the analyte was determined in tap water samples, and a satisfactory recovery rate was achieved.

## 1. Introduction

Currently, heavy metal contamination has become much more critical due to the increase in the Industrial Revolution. Lead (Pb) is a relatively abundant heavy metal, present in the Earth’s crust with an abundance of 0.0016% [1]. First used by humans as early as 3000 BC, Pb has been considered highly toxic for human health and the environment [2]. Pb is a hazardous non-biodegradable heavy metal. As Pb is smelted from ores and used in gasoline, batteries, pigments, etc., the resulting environmental pollution dramatically impacts ecosystems [3]. The lead ions in mines may cause water pollution when they are slowly released into the water environment through rainwater.

Additionally, it has been reported that lead ions can reach the human body through the gastrointestinal tract, respiratory tract, and skin and damage the nervous system, resulting in kidney, brain, and liver damage [4]. Moreover, lead ions in the human body in concentrations of 30–1000 µg L^−1^ can cause diseases such as atrophy, interstitial nephritis, colic, and anemia, among others [5]. The Drinking Water Quality Act criterion specified the value of 10 µg L^−1^ according to the guidelines established by the World Health Organization; therefore, it is harmful when the concentration of lead ions exceeds this value [6]. Considering the importance of the above, developing rapid and sensitive methods for detecting lead ions is crucial for analytical and environmental disciplines.

A large number of traditional analytical methods have been developed for the determination of lead ions, such as atomic absorption spectrometry [7], colorimetry [8], high-performance liquid chromatography (HPLC) [9], inductively coupled plasma analysis [10,11], mass spectrometry [12], fluorimetry [13,14], and electrochemistry [15], among others. Although these techniques for the detection of trace levels of Pb have their specific applicability and ability to improve the sensitivity, they exhibit typical shortcomings, including requiring expensive instruments, tedious operational procedures, relatively slow detection speeds, the specific design of responsive molecules, etc. Additionally, researchers are still seeking improvements in detection approaches, such as low cost, high sensitivity, simple operation, and ease of use. In comparison, electrochemical methods are more advanced, portable, and sensitive detection technologies, allowing rapid and simultaneous evaluations for lead ions [16].

Several lead ion electrochemical sensors focused on analyzing different surface modifiers have been developed in the past decade. Based on broad prospects for chemically modified electrodes and technological innovations toward advanced materials, developing a novel and effective approach for the electrochemical detection of lead ions has become a research hotspot. At present, studies on lead ion detection mainly focus on using various electrocatalysts, including metal–organic frameworks [17], carbon materials [18], polymers [19], bismuth-based materials [20], and functionalized DNA probes [21]. However, these materials do not meet the global demand for “carbon peaking” and “carbon neutrality” and may cause significant environmental damage. Therefore, it is necessary to develop low-cost and environmentally friendly electronic devices for lead ion detection [22].

Biochar (BC), a carbonaceous product from pyrolysis of biomass resources under a limited oxygen supply, has piqued research interest because of its environmentally friendly precursors [23]. Typically, a wide range of biomass industrial byproducts [24], plants [25], agricultural wastes [26], and forest residues [27] can be used cost-effectively for producing different types of biochar. Furthermore, biomass-derived biochar exhibits several distinctive characteristics, including chemical inertness, a sizable surface area, charged surfaces, and economic favorability. Consequently, it is applied in different fields such as heavy metal ion treatment [28], energy storage conversion devices [29], biomedicine [30], electrochemical sensors [31], photocatalysis [32], and others. Interestingly, these applications are mainly focused on an excellent adsorption capacity for positively charged metal ions because the biomass-derived biochar surface possesses a negative charge [33]. Previous studies indicated that physical and chemical interactions (electrostatic attraction, complexation, etc.) play critical functional roles in the adsorption mechanism [34]. Thus, surface modification has garnered significant attention to enhance raw biochar’s catalytic and absorption performances and obtain optimal composites.

Modifying biochar involves various techniques, including physical and chemical methods, to improve specific functionalities or properties. In recent times, acids [35], alkaline agents [36], metal oxides, and salts [37] have been utilized as chemical modifiers for the adsorption of heavy metal ions due to their high potential. Typically, metal oxides (Co_3_O_4_ [38], Fe_3_O_4_ [39], MoS_2_ [35], TiO_2_ [40]) have the potential to enhance affinity toward heavy metal contaminants because they can significantly improve the surface sorption, catalytic, and fixation abilities of biochar. Nanobiochar composites modified by metal oxides are an emerging material with exceptional quality. Among them, biochars modified with TiO_2_ nanomaterials are widely used as photocatalysts and adsorbents for heavy metal ions and organic pollutants [41,42]. In addition, TiO_2_ nanoparticles with different crystal planes have been applied to improve the catalytic effect of hydrogen absorption [43,44,45]. However, to the best of our knowledge, an electrochemical sensor based on a biochar–TiO_2_ nanocomposite has rarely been investigated. On the other hand, designing sustainable BC-based hybrid nanocomposites with appropriate surface functionalization is still challenging [46].

Herein, an electrochemical sensor was fabricated to analyze lead ions by modifying TiO_2_ nanoparticles on the surface of biochar (BC@TiO_2_NPs). Porous carbon materials were obtained by using spent coffee grounds. The prepared BC@TiO_2_NPs exhibited excellent electrical conductivity, which improved the adsorption capacity towards lead ions in aqueous solution. The influence of the experimental conditions on the lead ion detection was investigated. Finally, this work demonstrated that BC@TiO_2_NPs/GCE possessed good sensitivity for the detection of lead ions.

## 2. Results and Discussion

### 2.1. Detection Principle of the Sensor

**Scheme 1:**
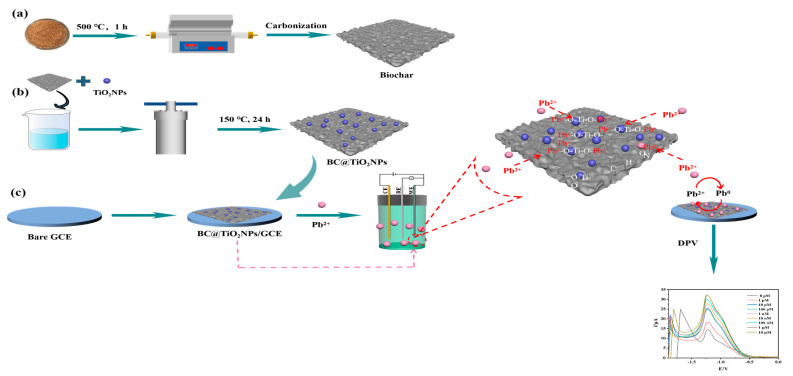
Schematic diagram of the application of BC@TiO_2_NPs nanomaterial and the detection principle of the electrochemical biosensor for lead ions. The inset in (c) is the photograph of the reaction mechanism of lead ions with the BC@TiO_2_NPs nanocomposite material.

This study introduced a rapid and sensitive detection method for Pb^2+^ based on a glass carbon electrode modified with a BC@TiO_2_NPs nanocomposite. The schematic of the BC@TiO_2_NPs nanohybrid synthesis and stepwise fabrication of the sensor is shown in Scheme 1. The BC was obtained using coffee grounds as the precursor by the thermal carbonization method (a). Then, TiO_2_ particles were randomly embedded into the BC network using an easy one-pot hydrothermal method (BC@TiO_2_NPs) (b). Next, the prepared BC@TiO_2_NPs composite was used to develop an efficient electrochemical sensing probe. When the probe was exposed to the Pb^2+^ solution, Pb^2+^ was first adsorbed onto the surface of the BC@TiO_2_NPs nanocomposite and then reduced to a low-valence state. This is because TiO_2_ nanoparticles can produce electron–hole pairs, and these carriers can redox-react with lead ions under an excitation voltage condition. Additionally, chelate complexes were formed on the surface of the BC@TiO_2_NPs nanocomposite owing to the empty orbital provided by lead ions and the lone pair electrons produced by the oxygen atoms in TiO_2_ nanoparticles (c). This greatly facilitated Pb^2+^ adsorption within the micropores and improved the sensitivity of the sensor towards Pb^2+^. Consequently, quantitative detection of Pb^2+^ was achieved by recording the electrochemical signals generated by the lead ion redox process.

### 2.2. Characterization of BC and BC@TiO_2_NPs Nanohybrid

**Figure 1:**
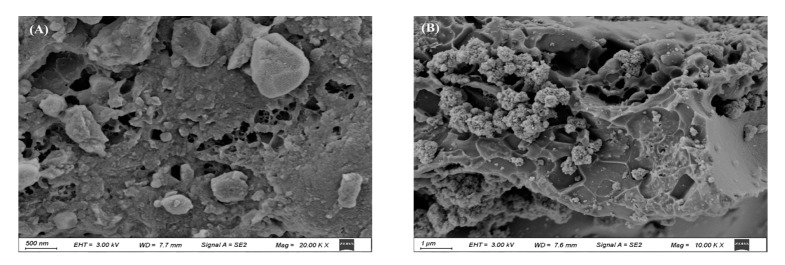
SEM images of BC (**A**) and BC@TiO_2_NPs (**B**).

The BC@TiO_2_NPs were prepared using biochar obtained from coffee grounds as the precursor. A scanning electron microscope (SEM) was employed to inspect the morphology and microstructure of the as-prepared BC and BC@TiO_2_NPs materials. As illustrated in Figure 1A, the BC showed a blocky porous structure that can facilitate rapid ion transport, and the porosity was roughly calculated as 4.207%, according to the ratio of the porous area to the total area of the SEM image (Table S1). As seen from Figure 1B, small-sized TiO_2_ nanoparticles (average 0.5218 µm in Figure S1A) were randomly self-assembled on the surface of the BC composite through the interaction between the BC and TiO_2_. Additionally, we provide real sample images of BC and BC@ TiO_2_NPs materials in Figure S1B. The results showed a distinct color change between the two materials. Consequently, these results indicated the successful preparation of the BC@TiO_2_NPs nanohybrid composite, which can be used for the electrochemical sensor construction.

### 2.3. Electrochemical Behavior of Modified Electrodes

**Figure 2:**
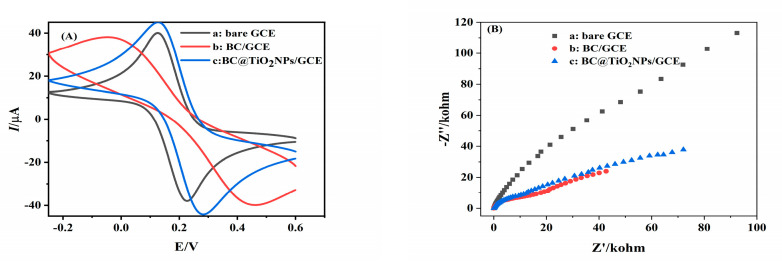
Characterization of the Pb^2+^ electrochemical sensor. CV (**A**) and EIS (**B**) of differently assembled electrodes: bare GCE (a), BC/GCE (b), BC@TiO_2_NPs/GCE (c); EIS and CV were operated in 0.1 M KCl containing 5 mM [Fe (CN)_6_]^3−^/^4−^ with the frequency range from 0.1 Hz to 100 kHz, the amplitude of 10 mV, and the scanned potential from -0.2 V to +0.6 V, with a scan rate of 0.05 V/s.

The electrochemical properties of the modified electrode were characterized by cyclic voltammetry (CV) and electrochemical impedance spectroscopy (EIS) using 5.0 mM K_3_[Fe(CN_6_)]^3−/4−^ containing 0.1 M KCl as an electrolyte. The cyclic voltammograms and the Nyquist plots of bare GCE, BC/GCE, and BC@TiO_2_/GCE are shown in Figure 2. As seen in Figure 2A, the bare GCE produced a pair of well-defined redox peaks (curve a), ascribed to the oxidation and reduction reactions between [Fe(CN_6_)]^3−/4−^ ions. However, after BC material modification (BC/GCE), there was a significant reduction in the redox peak (curve b) because of the constraint of the electron transfer toward redox reactions caused by the porous BC with poor electronic conductivity. Notably, after introducing TiO_2_NPs onto the surface of the BC material to form BC@TiO_2_/GCE (curve c), the redox peak significantly increased, which could be attributed to parts of TiO_2_ nanoparticles being electroactive and improving the charge transfer ability. This also confirms the successful decoration of BC and BC@TiO_2_ onto the surface of GCE. These results were consistent with the corresponding EIS characterizations (Figure 2B), in which the electron transfer resistance varied for the differently assembled electrodes. The semicircular diameter of the impedance equaled the electron transfer resistance (Ret) at the electrode interface, which confirmed the electrode modification process.

### 2.4. Feasibility Validation of the Electrochemical Sensor

**Figure 3:**
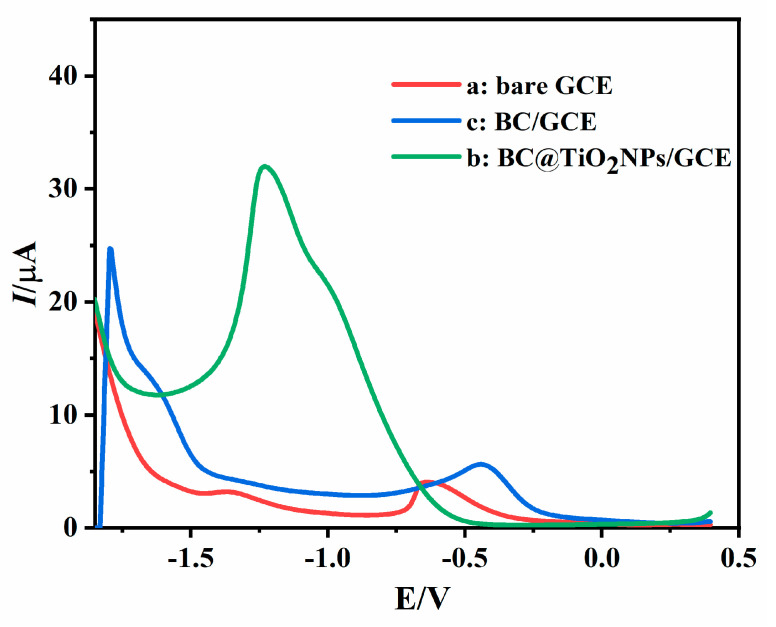
DPV responses of the fabricated BC@TiO_2_NPs sensor in 0.1 M NaAc-HAc solution (pH 4.5) with bare GCE (a), BC/GCE (b), and BC@TiO_2_NPs/GCE (c). Concentration of lead ions 10 µM. Modulation time of 0.02 s; interval time of 0.5 s; pulse amplitude of 25 mV.

To ensure the success of the subsequent experiment and investigate the electrochemical response of Pb^2+^ by the GCE, BC/GCE, and BC@TiO_2_NPs/GCE sensors, a differential pulse voltammetry (DPV) experiment was performed in 0.1 M NaAc-HAc buffer (pH = 4.5) containing 10 µM lead ions. As depicted in Figure 3, in the presence of bare GCE, there was almost no peak current at −0.80 V (curve a). In contrast, the modified electrode (BC@TiO_2_/GCE) exhibited a well-defined oxidation peak current response (curve b), three times higher than that of bare GCE and BC/GCE. The porous structure of BC causes the cationic lead to be adsorbed in the pores. Subsequently, the lead ion is electrochemically reduced to zero valence under the applied voltammetry potential and then oxidized to generate an anodic peak current. These results show that the TiO_2_NPs decoration on the BC results in a synergy that leads to a notable improvement in the surface area, electronic conductivity, and active functional sites. However, a distinct decrease in the peak current was observed when the BC was immobilized on the bare GCE (curve c). This decrease in the current value indicates that the unmodified biochar can hinder the diffusion of the redox species toward the electrode surface from the electrolyte. These results confirm the efficiency of the designed strategy for detecting lead ions.

### 2.5. Optimization of Conditions

In order to achieve the best experimental conditions for detecting lead ions, the key factors were optimized, including the type and pH of the electrolyte, concentration of BC@TiO_2_NPs, and deposition time.

#### 2.5.1. Effect of Electrolyte and pH

Supporting electrolytes maintain a homogeneous electric field in electrochemical investigations involving the oxidation and reduction of the analyte molecule. As shown in Figure S2, the current signal and potential peak of the analyte are significantly influenced by various electrolytes, including phosphate acid buffer solution (PBS), acetic acid buffer solution (NaAc-HAc), and ammonium chloride buffer solution (NH_3_H_2_O-NH_4_Cl). The results revealed that the NaAc-HAc buffer displayed the maximum peak current (Ipa). Consequently, the NaAc-HAc buffer was considered the optimal supporting electrolyte for the further detection of lead ions. Next, the effect of pH on the lead ion response in NaAc-HAc buffer was evaluated. The DPV response of BC@TiO_2_NPs/GCE sensor was recorded in various pH values (4.0–6.5) of NaAc-HAc buffer. The current response reached the highest value when the solution pH was 4.5 (Figure S3), probably because overly acidic or basic solutions affect the target’s molecular structure. Therefore, the following sensing process was carried out under pH = 4.5.

#### 2.5.2. Effect of Concentration of BC@TiO_2_NPs Composite

The concentration of the BC@TiO_2_NPs composites during the electrocatalytic process directly affected the electrochemical performance for determining lead ions. As seen in Figure S4, when the BC@TiO_2_NPs concentration increased from 2 mg/mL to 12 mg/mL, the oxidation current intensity increased and reached its maximum value at 10 mg/mL. The porous structure of BC@TiO_2_NPs composite offers electroactive sites at the electrolyte−electrode interface. However, overloading can affect the thickness of this composite, hindering lead ion diffusion and increasing electron transport resistance. Thus, 10 mg/mL BC@TiO_2_NPs was utilized for the following detection experiments.

#### 2.5.3. Effect of Deposition Time

The effect of the deposition time of lead ions on BC@TiO_2_NPs/GCE was studied within the range of 1 to 12 s (Figure S5). It was observed that the current value exhibited a gradual increase with the extension of the deposition time up to 3 s. Hence, an adsorption time of 3 s was selected for practical purposes in the subsequent experiment.

### 2.6. Analytical Performance of the Proposed Method

**Figure 4:**
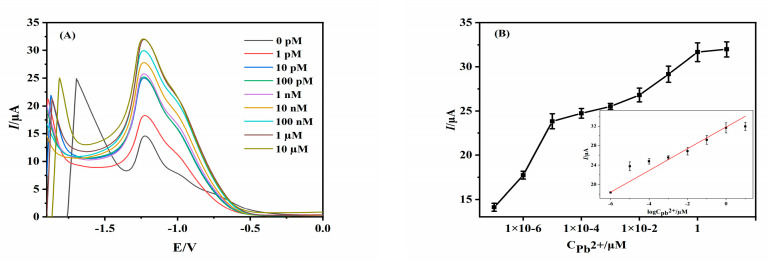
(**A**) Electrochemical responses of the BC@TiO_2_NPs/GCE sensor to different concentrations of Pb^2+^ from 0 pM to 10 µM recorded in 0.1 M NaAc-HAc solution (pH 4.5). (**B**) Calibration plots between the current and the logC_Pb2+_. Error bars, SD, *n* = 3.

Under the optimized experimental conditions (pH = 4.5, NaAc-HAc buffer, 10 mg/mL BC@TiO_2_NPs, 3 s deposition time), DPV was employed to detect Pb^2+^ adsorption behavior on BC@TiO_2_NPs/GCE. As depicted in Figure 4 A, the oxidation peak current (*I*_pa_) increased with the increase in Pb^2+^ concentration. *I*_pa_ presented a well-defined linear relationship with the logarithm of Pb^2+^ concentration in the 1 pM-10 µM range. The corresponding linear regression equation was expressed as *I* (μA) = 2.23541logC_Pb2+_ + 31.8198 (R^2^ = 0.9667), and the detection limit was determined to be 0.6268 pM according to the calibration curve method. The calculation formula of LOD is LOD = 3σ/S, where σ is the standard deviation of the detection value of blank samples, and S is the slope of the calibration curve. This result indicated that more lead ions were adsorbed onto the cavities of the porous biochar owing to abundant binding sites at the BC@TiO_2_NPs nanocomposite. Compared with previously reported electrochemical sensors for lead ions (Table 1), the proposed BC@TiO_2_NPs-modified electrode presented better analytical performance. Therefore, the fabricated sensor significantly improved the detection sensitivity toward lead ions.

### 2.7. Selectivity, Reproducibility, and Stability Investigation

**Figure 5:**
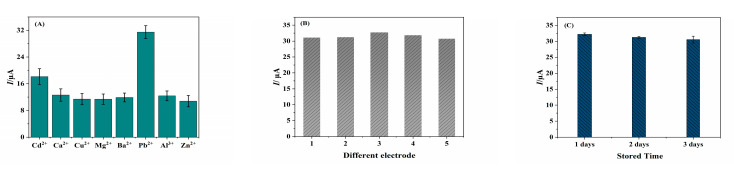
(**A**) High selectivity of the designed sensor for Pb^2+^ detection. One µM of Pb^2+^ and various ions, including Cd^2+^, Ca^2+^, Cu^2+^, Mg^2+^, Ba^2+^, Al^3+^, and Zn^2+^ (100 µM), were used in the experiments. (**B**) Reproducibility of the Pb^2+^ electrochemical sensor was evaluated by measuring 10 µM of lead ions with different electrodes. (**C**) Stability evaluated by the BC@TiO_2_NPs/GCE sensor in the presence of lead ions.

To assess the selection accuracy of the prepared BC@TiO_2_NPs sensor, the selectivity was estimated using 100 µM solutions of different cations (Cd^2+^, Ca^2+^, Cu^2+^, Mg^2+^, Ba^2+^, Al^3+^, Zn^2+^). The results revealed that the current signal of Pb^2+^ ions exhibited a maximum value (Figure 5A). However, the sensor’s response to different ions (Cd^2+^, Ca^2+^, Cu^2+^, Mg^2+^, Ba^2+^, Al^3+^, Zn^2+^) was almost equivalent, which may mean that changes in the ionic strength can affect the electrode response. In addition, the interfering ions may react chemically with the electrode membrane, resulting in a change in the properties of the electrode membrane, which then produces a potential response. In general, the proposed method based on the BC@TiO_2_NPs composites possessed good specificity for Pb^2+^ detection.

To evaluate the reproducibility of the sensor, further investigations were conducted by preparing five BC@TiO_2_NPs/GCE sensors using the same modification process and testing them with 10 μM lead ions in NaAc-HAc buffer solution (pH = 4.5). It was observed that the peak current of lead ions was roughly the same (Figure 5B), and the relative standard deviation (RSD) value was found to be 2.50% (*n* = 5), indicating good repeatability of the developed sensor. In addition, one independent BC@TiO_2_NPs/GCE electrode was also used to test the reproducibility experiment under same conditions. The results are shown in Figure S6A, and the RSD was calculated as 14.8%, which indicated that the BC@TiO_2_NPs/GCE can operate under different lead ion concentrations.

Stability is also crucial in assessing the sensor’s performance and service life. Here, 10 mg/mL of BC@TiO_2_NPs-altered GCE was stored for 1 day, 2 days, and 3 days at room temperature, respectively. Furthermore, the DPV signals are shown in Figure 5C, and there were fewer changes in the observed current signals for each measurement, demonstrating commendable storage stability with an RSD of 2.70%. To estimate the lifetime of BC@TiO_2_NPs/GCE electrode, cyclic experiments were carried out via cyclic voltammetry (CV) technology (Figure S6B). The results shown that as the number of cycles increased, the oxidation peak current of lead ions gradually enhanced, possibly due to the electro-adsorption and desorption reactions of lead ions on the BC@TiO_2_NPs/GCE electrode surface. The capacity retention rate of the sensor was about 116.0% even after 50 cycles according to the formula: Capacity Retention Ratio = *I*_n_/*I*_0_, where *I*_n_ is the peak current value after 50 cycles, and *I*_0_ is the initial peak current value, indicating that the sensor had excellent storage capacity.

### 2.8. Analysis of Water Samples

To evaluate the practical performance of the fabricated BC@TiO_2_NPs/GCE sensor, it was applied to analyze the content of lead ions in a natural water sample. Initially, the quantification of lead ions was conducted through the standard addition method, and different concentrations of lead ions (1, 10, and 100 nM) were spiked into tap water samples, respectively. Next, the current response was determined using the DPV technique under the same conditions. As presented in Table 2, the recovery rates of lead ions from the water samples ranged between 100.09 and 103.0% with RSD values from 0.8792 to 1.0910%, suggesting the high potential of the BC@TiO_2_NPs/GCE sensor for the reliable determination of lead ions in the actual water samples.

## 3. Materials and Methods

### 3.1. Reagent and Apparatus

Waste coffee grounds were purchased from Arabica-Cardim Coffee in Nandu River, Pu’er, Yunnan province. Titanium dioxide (TiO_2_), ammonia water (NH_3_ H_2_O), ethyl alcohol (CH_3_CH_2_OH, 95%), glacial acetic acid (CH_3_COOH), and sodium anhydrous acetate (CH_3_COONa) were provided by Shanghai Titan Scientific Co., Ltd. (Shanghai, China). Sodium dihydrogen phosphate dihydrate (NaH_2_PO4 2H_2_O), disodium hydrogen phosphate dodecahydrate (Na_2_HPO_4_ 12H_2_O), potassium hexacyanoferrate (III) (K_3_Fe(CN)_6_), and potassium hexacyanoferrate (II) (K_4_Fe(CN)_6_ 3H_2_O) were purchased from Tianjin Damao Chemicals Reagent Factory (China). Anhydrous sodium sulfate (Na_2_SO_4_) was supplied by Tianjin Zhiyuan Chemicals Reagent Co., Ltd. (Tianjin, China). Lead chloride (PbCl_2_) was obtained from Shanghai Reagent Factory No.1 Co., Ltd. (Shanghai, China), and solid potassium chloride (KCl) was purchased from Sichuan Xilong Science Co., Ltd. (Sichuan, China). For evaluation of the sensor performance, calcium (Ca^2+^), cadmium (Cd^2+^), copper (Cu^2+^), and magnesium (Mg^2+^) standard solutions were acquired from The National Center for Analysis and Testing of Nonferrous Metals and Electronic Materials. All solutions were prepared using ultrapure water.

Field emission scanning electron microscopy (SEM) images of the BC@TiO_2_NPs nanohybrid were obtained using a German Zeiss Sigma 300 microscope. Cyclic voltammetry (CV), differential pulse voltammetry (DPV), and electrochemical impedance spectroscopy (EIS) measurements were performed using a CHI660C electrochemical workstation (ChenHua Instrument Co., Shanghai, China) at room temperature. A typical three-electrode system, composed of a bare or modified glass–carbon electrode (GCE) as the working electrode, a saturated calomel electrode (SCE) as the reference electrode, and a platinum wire as the auxiliary electrode, was used for electrochemical measurements.

### 3.2. Preparation of BC@TiO_2_NPs Nanocomposite

The BC@TiO_2_NPs nanocomposite was prepared using the following two steps. First, the coffee grounds were boiled five times in ultrapure water and then dried at 80 °C for three days. To obtain biochar (BC), the dried coffee grounds were carbonized at 500 °C for 1 h in a tube furnace under a nitrogen atmosphere (10 °C/min). After screening with a 0.125 mm sieve, the pyrolyzed coffee BC material was stored in sealed bottles for further use. Afterwards, 2.1 g of pyrolyzed BC material and 0.9 g of TiO_2_ nanoparticles were mixed in a Teflon-lined stainless-steel autoclave with 30 mL of ultrapure water. The mixture was then heated to 150 °C for 24 h. Finally, the resulting product was centrifuged and rinsed with Milli-Q water and ethyl alcohol repeatedly. After that, the obtained sample was dried at 60 °C for 24 h and named BC@TiO_2_NPs.

### 3.3. Fabrication of Electrochemical Sensor for Lead Ions

Before preparing the modified electrode, a GCE was initially polished with 0.3 and 0.05 μm Al_2_O_3_ powder to improve its surface kinetics. Then, it was ultrasonically cleaned with ethanol and ultrapure water for 1 min, respectively. Next, 10 mg of BC@TiO_2_NPs nanocomposite was dispersed in 1 mL of ultrapure water (10 mg/mL) and treated in a sonicator for 2 h; then, 10 μL of the uniform solution was withdrawn and dripped onto the surface of the smooth and polished GCE, followed by drying naturally in air at 25 °C for 1 h. The modified electrode served as a working electrode for electrochemical detection.

### 3.4. Electrochemical Measurement

The electrochemical performance of the fabricated GCE was determined by cyclic voltammetry (CV) and electrochemical impedance spectra (EIS) measurements. The CV and EIS experiments were conducted using 5 mM [Fe (CN)_6_]^3−^/^4−^ electrolyte containing 0.1 M KCl over the voltage range of −0.2–0.6 V, at a scan rate of 0.05 V/s, a frequency range of 0.1 Hz to 100 kHz, and a potential amplitude of 10 mV. Differential pulse voltammetry (DPV) curves were constructed using 0.1 M NaAc-HAc buffer (pH = 4.5) as the electrolyte with the potential between −2.0 and 0.6 V, at a pulse time of 0.02 s, pulse period of 0.5 s, and amplitude of 25 mV.

### 3.5. Optimization of Experimental Conditions

In order to achieve the best experimental conditions for detecting lead ions, this experiment further studied the influence of possible factors, including the type of buffer solution, pH, modification concentration of BC@TiO_2_NPs nanocomposite, and deposition time. The selection of these experimental conditions was achieved using DPV technology, with specific details described in Section 2.5.

### 3.6. Determination of Lead Ions

Lead ions were detected using DPV technology under the optimal experimental conditions presented in Section 3.5. First, 40 mL of 0.1 M NaAc-HAc buffer solution with a pH of 4.5 was taken in beaker, and an appropriate amount of lead standard solution was added to prepare eight standard solutions with different concentrations of 1 pM, 10 pM, 100 pM, 1 nM, 10 nM, 100 nM, 1 μM, and 10 μM. Then, the pretreated three-electrode system was inserted into the electrolysis tank and connected to the electrochemical workstation. Referring to Section 3.4, the parameters of the DPV were set, and a scan was performed in the potential range of −2 V to 0.6 V. The peak current values corresponding to lead ions in the DPV curves were recorded, and a standard curve was plotted with the lead ion concentration as the horizontal coordinate and the peak current value as the vertical coordinate. The experimental result in Section 2.6 is based on this step.

### 3.7. Real Sample Tests

To investigate the practical application of the proposed sensor in routine analysis, BC@TiO_2_/GCE was employed to detect Pb^2+^ in Pu’er water samples. First, local water samples from Pu’er City were treated using a membrane filter with a pore size of 0.45 μm, and the pH of the water samples was adjusted to 4.5 using a 0.1 M NaAc-HAc buffer solution. A precise volume of 40 mL of the pretreated actual water sample was then placed in a beaker, and known concentrations of lead ions within the linear range of the standard curve, such as 1, 10, and 100 nM, were added as standard solutions. According to the electrochemical detection conditions in Section 3.6, the spiked water samples were tested to obtain the desorption peak current. Based on the linear regression equation of the standard curve, the concentration of spiked lead ions was calculated, and the recovery rate was determined.

## 4. Conclusions

A simple, green, and sensitive BC@TiO_2_NPs-modified working electrode was successfully fabricated and applied to detect lead ions. First, a BC@TiO_2_NPs nanocomposite derived from coffee residue was synthesized and characterized using SEM, CV, and EIS techniques. Then, TiO_2_ nanoparticles were successfully dispersed throughout the surface of the mesoporous BC, which enormously improved the peak currents for lead ion determination. The resulting electrode demonstrated outstanding sensitivity, a low limit of detection (0.6268 pM), a wide linear range (1 pM–10 µM), good interference, good repeatability, and practical stability. Additionally, this sensor was employed to monitor lead ions in real samples and displayed remarkable recovery values (100.09–103.0%). Therefore, the results indicate that carbon materials derived from waste coffee residues can be utilized to manufacture high-value devices. Additionally, the BC@TiO_2_NPs/GCE sensor has an expectation for serving as a reliable electrochemical platform in the field of environmental monitoring, demonstrating significant potential for reducing carbon footprints.

## Figures and Tables

**Table 1:** Comparisons of the proposed electrochemical sensor with the reported methods.

Strategy	Technique	Linear Range/μM	LOD/μM	Reference
G-C-4/SPE	SWASV	0.05–1	0.0089	[1]
PA-PPy@SPCE	DPASV	0.01–6	0.43 × 10^−3^	[47]
CHO/CS-GO/Pb(II)/Aptmer/MCH/AuNPs/GCE	DPV	0.483 × 10^−6^–0.965 × 10^−5^	0.384 × 10^−6^	[48]
BC@TiO_2_NPs/GCE	DPV	1 × 10^−6^–10	0.6268 × 10^−6^	This method

**Table 2:** Detection of Pb^2+^ in an actual water sample (*n* = 3).

Sample (Tap Water)	Added (nM)	Found (nM)	Recovery (%)	RSD (%)
1	0	0	0	0
2	1	1.030	103.00	0.9324
3	10	10.046	100.46	0.8792
4	100	100.088	100.088	1.0910

## Data Availability

The data presented in this study are available in the article and can be shared upon request.

## Supplementary Materials

The following supporting information can be downloaded at: https://www.mdpi.com/article/10.3390/molecules29235704/s1, Figure S1: (A) The particle size distribution histogram of TiO_2_ nanoparticles. (B) The real samples of BC and BC@TiO_2_NPs; Figure S2: (A) Effects of various electrolytes including PBS, NaAc-HAc, NH_3_H_2_O-NH_4_Cl. (B) The error bars represent the standard deviation of three independent experiments, *n* = 3; Figure S3: Effects of the pH on the sensor in the NaAc-HAc buffer. The error bars represent the standard deviation of three independent experiments, *n* = 3; Figure S4: Effects of the BC@TiO_2_NPs concentration. The error bars represent the standard deviation of three independent experiments, *n* = 3; Figure S5: Effects of the deposition time on the Pb^2+^ detection. The error bars represent the standard deviation of three independent experiments, *n* = 3; Figure S6 (A) DPV responses of one BC@TiO_2_NPs/GCE electrode for detecting lead ions at different concentrations including 1 pM, 10 pM, 100 pM, 1 nM, 10 nM in 0.1 M NaAc-HAc buffer (pH 4.5). (B) Cyclic voltammograms of 10 μM of lead ions recorded at the BC@TiO_2_NPs/GCE sensor in 0.1 M NaAc-HAc of pH 7.0 at a scan rate of 0.05 V/s, 50 cycles, and a potential range of -1.80 to 0.4 V; Table S1: Porosity data of BC material.
