# Supplementary material for: An Electrochemical Sensor for Detection of Lead (II) Ions Using Biochar of Spent Coffee Grounds Modified by TiO2 Nanoparticles
Source: Molecules. 2024 Dec 3;29(23):5704. doi: 10.3390/molecules29235704 (PMC11643534; doi:10.3390/molecules29235704)
Supplement: Supplementary file 1 [file molecules-29-05704-s001.zip › molecules-3279126-supplementary.pdf]

Supplementary Information

An electrochemical sensor for detection of lead (II) ions using biochar of spent coffee grounds modified by TiO_2 nanoparticles

Zaiqiong Liu [1], Yiren Xu [1], Xurundong Kan [1], Mei Chen [1], Jingyang Dai [1], Yanli Zhang [2], Pengfei Pang [2], Wenhui Ma [1,3,*] and Jianqiang Zhang [1,*]

[1] International Union Laboratory of China and Malaysia for Quality Monitoring and Evaluation of Agricultural Products in Yunnan, School of Biology and Chemistry, Pu'er University, Pu'er 665000, China; lzq15025110725@126.com (Z.L.); xuyiren@peu.edu.cn (Y.X.); kanxurundong@163.com (X.K.); mchen1988@126.com (M.C.); (J.D.);

[2] School of Chemistry and Environment, Yunnan Minzu University, Kunming 650500, China; yanli.zhang@yahoo.com (Y.Z.); pfpang@ynni.edu.cn (P.P.)

[3] Yunnan University, Kunming 650500, China

* Correspondence: mwhsilicon@126.com (W.M.); drjqzhang@126.com (J.Z.); Tel./Fax: +86-15348798859 (J.Z.)

Supporting figure captions:

Fig. S1 (A) The particle size distribution histogram of TiO_2 nanoparticles. (B) The real samples of BC and BC@TiO_2NPs.

Fig. S2 (A) Effects of various electrolytes including PBS, NaAc-HaC, NH_3H_2O-NH_4Cl. (B) The error bars represent the standard deviation of three independent experiments, *n*=3.

Fig. S3 Effects of the pH on the sensor in the NaAc-HAc buffer. The error bars represent the standard deviation of three independent experiments, *n*=3.

Fig. S4 Effects of the BC@TiO_2NPs concentration. The error bars represent the standard deviation of three independent experiments, *n*=3.

Fig. S5 Effects of the deposition time on the Pb^{2+} detection. The error bars represent the standard deviation of three independent experiments, *n*=3.

Fig. S6 (A) DPV responses of one BC@TiO_2NPs/GCE electrode for detecting lead ions at different concentrations including 1 pM, 10 pM, 100 pM, 1 nM, 10 nM in 0.1 M NaAc-HAc buffer (pH 4.5). (B) Cyclic voltammograms of 10 μM of lead ions recorded at the BC@TiO_2NPs/GCE sensor in 0.1 M NaAc-HAc of pH 7.0 at a scan rate of 0.05 V/s, 50 cycles, and a potential range of -1.80 to 0.4 V.

Table S1 Porosity data of BC material.

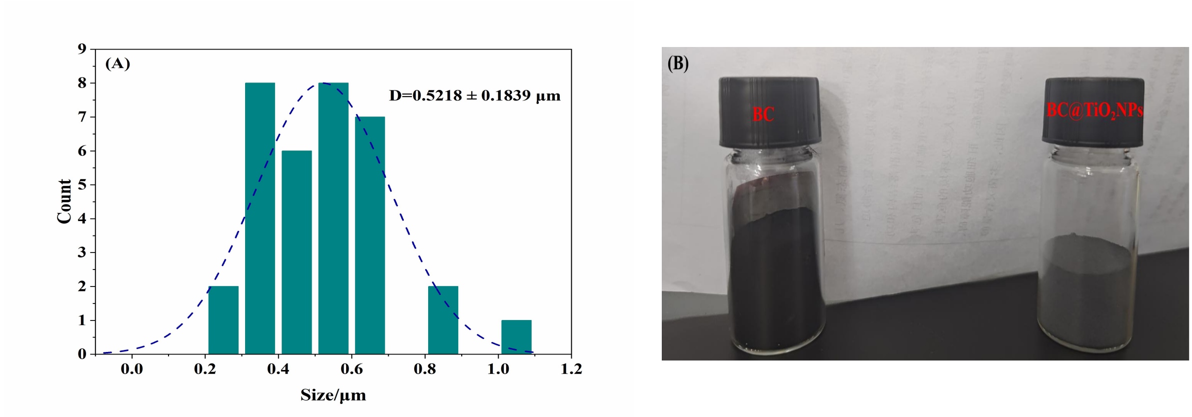

Fig. S1 (A) The particle size distribution histogram of TiO_2 nanoparticles. (B) The real samples of BC and BC@TiO_2NPs.

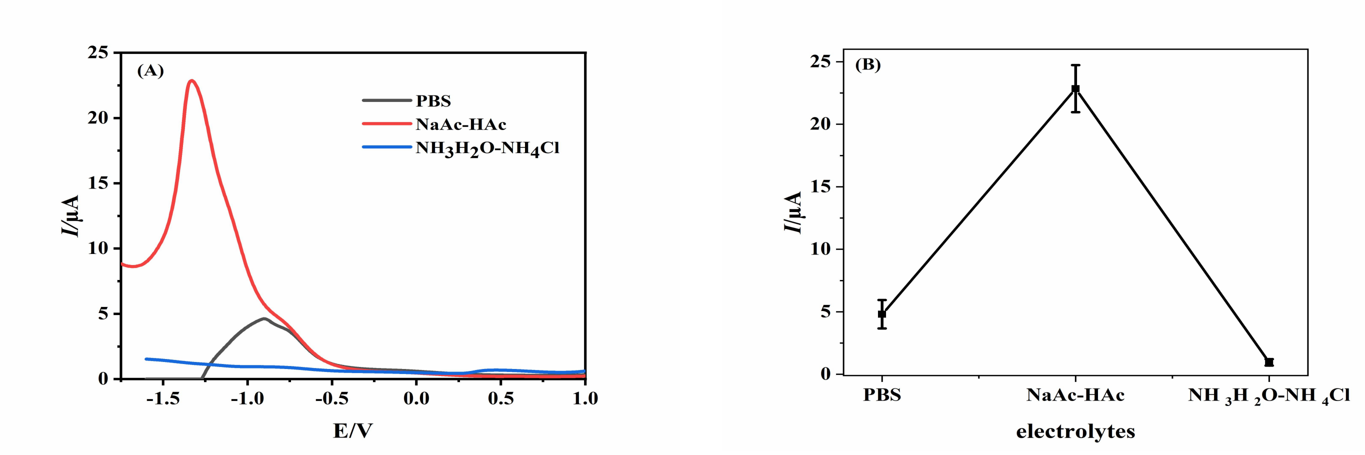

Fig. S2 (A) Effects of various electrolytes including PBS, NaAc-HaC, NH_3H_2O-NH_4Cl. (B) The error bars represent the standard deviation of three independent experiments, *n*=3.

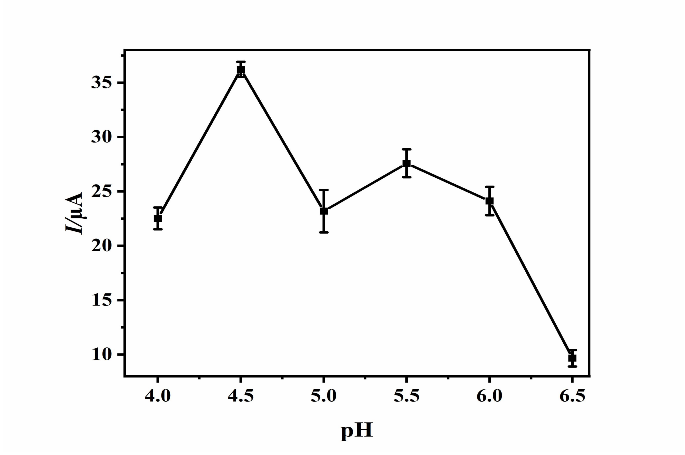

Fig. S3 Effects of the pH on the sensor in the NaAc-HAc buffer. The error bars represent the

standard deviation of three independent experiments, *n*=3.

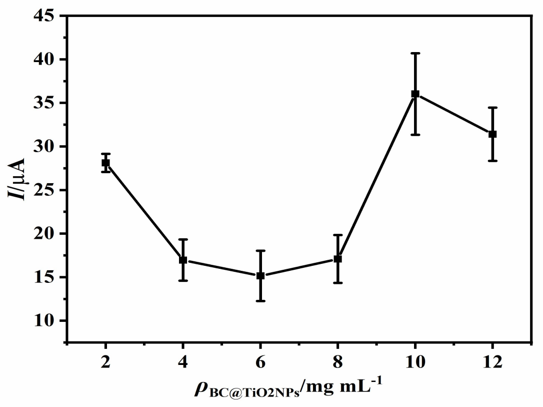

Fig. S4 Effects of the BC@TiO2NPs concentration. The error bars represent the standard deviation of three independent experiments, *n*=3.

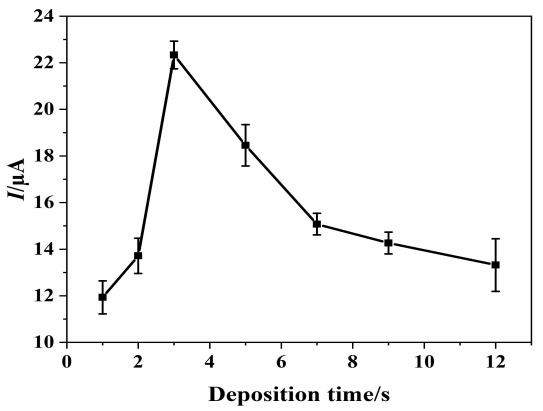

Fig. S5 Effects of the deposition time on the Pb^{2+} detection. The error bars represent the standard deviation of three independent experiments, *n*=3.

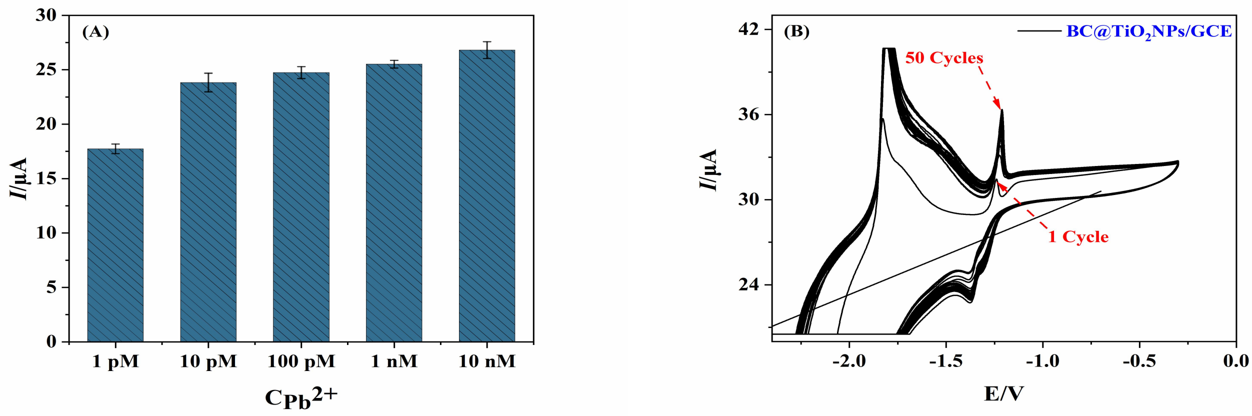

Fig. S6 (A) DPV responses of one BC@TiO2NPs/GCE electrode for detecting lead ions at different concentrations including 1 pM, 10 pM, 100 pM, 1 nM, 10 nM in 0.1 M NaAc-HAc

buffer (pH 4.5). (B) Cyclic voltammograms of 10 μM of lead ions recorded at the BC@TiO_2NPs/GCE sensor in 0.1 M NaAc-HAc of pH 7.0 at a scan rate of 0.05 V/s, 50 cycles, and a potential range of -1.80 to 0.4 V.

Table S1 Porosity data of BC material.

Count	Total Area	Average Size	Porosity/%
441	33083	75.018	4.207
